# MEMORY ISSUES IN RETROSPECTIVE LIFE HISTORY SURVEYS: DO QUESTION TYPE AND TOPIC MATTER?

**DOI:** 10.1093/geroni/igae098.3193

**Published:** 2024-12-31

**Authors:** Wenqing Qian, Wenshan Yu, Mary Beth Ofstedal, Jacqui Smith, Marina Larkina, Amanda Sonnega

**Affiliations:** University of Michigan, Ann Arbor, Michigan, United States; Duke University, Ann Arbor, Michigan, United States; University of Michigan, Ann Arbor, Michigan, United States; University of Michigan, Ann Arbor, Michigan, United States; University of Michigan, Ann Arbor, Michigan, United States; University of Michigan, University of Michigan, Michigan, United States

## Abstract

Collecting retrospective information from the older population is critical yet challenging, and especially in self-administered surveys. Older adults may have difficulty retrieving autobiographical memories, navigating skip patterns in mail surveys, or using the complex technology often required for web surveys. These factors may lead to potential data quality concerns. Our study investigates data quality in the Health and Retirement Study (HRS) Life History Mail Survey (LHMS: N = 14934), that asks people over age 50 about events and experiences during childhood and midlife. We investigate characteristics of survey items that are related to item-missingness. Items were coded on 4 dimensions: (1) format: whether it was part of a grid or not, (2) life period: childhood vs. midlife, (3) detail: semantic vs. episodic, and (4) topic: residence, education, work, partnership, health. Overall, the item missing rate (IMR) was low (7.8%). Grids had higher IMRs than non-grid items (14.2% vs 6.4%). Controlling for this, we found that the average IMR for midlife questions was higher than for those pertaining to childhood in non-grid formats (8.9% vs. 4.9%), but similar in grid formats (11.6% vs. 12.2%). Additionally, episodic questions had a higher IMR compared to semantic questions in grid formats (13.7% vs. 10.3%), but lower in non-grid formats (3.1% vs. 6.1%). Among the five sections, items related to work and health history exhibited higher IMRs for both grid and non-grid formats. We discuss the implications of these results for the design of self-administered surveys and for analyses based on data from the LHMS.

